# Digital phenotyping and data analysis for plant breeding

**DOI:** 10.1270/jsbbs.72.1

**Published:** 2022-03

**Authors:** Sachiko Isobe, Seishi Ninomiya

**Affiliations:** 1 Department of Frontier Research and Development, Kazusa DNA Research Institute; 2 Graduate School of Agriculture and Life Sciences, The University of Tokyo; 3 Plant Phenomics Research Center, Nanjing Agricultural University

The life sciences have entered an era of big data analysis over the last decade. This is mainly due to the large-scale acquisition of genome information by the advent of next generation sequencing technologies and the development of data analysis technologies such as artificial intelligence. Digital technology has also been developed in plant phenotyping and has begun to be introduced into crop breeding. In contrast to genome sequencing, a variety of measurement technologies are required in plant phenotyping depending on the target traits and plants. In addition, the analysis methods for the acquired data are still in the process of development, and it is difficult to choose the best method without sufficient knowledge.

Therefore, this special issue features the current status of digital plant phenotyping technology and data analysis methods. There are five review and five research articles included in this issue. The first review article gives an overview of the current status and prospects of high-speed phenotyping technology for crops. The second article describes ways of using morphometric descriptors to represent morphological traits. The third article reviews the creation of 3D models, which is one of the most popular aspects of digital phenotyping. The fourth article reviews the available technologies for measuring roots, which is one of the most challenging traits in plant phenotyping. The fifth article is a review of metabolomics analysis, since chemical component analysis is another important part of phenotyping. The sixth to tenth articles are research papers describing the actual technology development for digital phenotyping or data analysis of plants, including the development of data acquisition equipment and methods for extracting necessary information through image analysis.

The development of digital plant phenotyping technology has been driven by the convergence of biological, informatics, and engineering research fields. Many of the papers in this special issue are written by authors who are involved in engineering or information science rather than breeding science. Thus, there may be unfamiliar words that are difficult to read for the typical readers of BS. Despite this unfamiliarity, we hope that this special issue will be read by many BS readers, and will provide an opportunity to enter this new research field.

